# Development of the Method for Nusinersen and Its Metabolites Identification in the Serum Samples of Children Treated with Spinraza for Spinal Muscular Atrophy

**DOI:** 10.3390/ijms231710166

**Published:** 2022-09-05

**Authors:** Sylwia Studzińska, Maria Mazurkiewicz-Bełdzińska, Bogusław Buszewski

**Affiliations:** 1 Environmental Chemistry and Bioanalytics, Faculty of Chemistry, Nicolaus Copernicus University in Toruń, 7 Gagarin Str., PL-87-100 Toruń, Poland; 2 Department of Developmental Neurology, Medical University of Gdansk, 7 Dębinki Str., PL-80-952 Gdańsk, Poland

**Keywords:** nusinersen, oligonucleotides, ion pair chromatography, mass spectrometry, extraction, metabolites

## Abstract

The application of oligonucleotides as drugs for different genetic diseases is increasing rapidly. Since 2016 they are used during spinal muscular atrophy treatment with the use of nusinersen oligonucleotide. The purpose of this study was to improve methods for the analysis of serum samples of patients treated with nusinersen. The results showed that liquid-liquid extraction (with phenol/chloroform) is insufficient and an additional purification step using solid-phase extraction is necessary. The best results were obtained for microextraction by packed sorbents. Important parameters in the optimization of the method were mainly the type of amine in the mobile phase and the stationary phase. Both influenced the selectivity of metabolite separation and thus their correct identification; while amine type impacted also the intensity of signals. Finally, the highest resolution of separation and the highest peak areas were obtained for *N*,*N*-dimethylbutylamine or *N*,*N*-diisopropylthylamine with an octadecyl column with a terminal aryl group. Over a dozen of metabolites were successfully identified with the use of methods developed during the study. The 3′ exonucleases and 5′ exonucleases were mainly responsible for nusinersen metabolism, consequently, 3′end shortmers, and 5′end shortmers were observed, as well as metabolites with simultaneous loss of bases at both ends of the sequence. However, some depurination and depyrimidination products were also identified. To the best of our knowledge, this is the first report on nusinersen and its metabolite identification in serum samples by liquid chromatography and mass spectrometry.

## 1. Introduction

The application of oligonucleotides (OGNs) for different biomedical applications is increasing rapidly. Antisense therapy is one such example [1]. Although research on this type of therapy has been ongoing for many years, a breakthrough has been observed in the last six years, when the FDA approved as many as 14 drugs to treat various types of genetic diseases, such as spinal muscular atrophy (SMA) [1,2]. It is a rare genetic disease that causes progressive weakness and loss of muscle strength. Unfortunately, most children with SMA do not survive beyond two years of age. There are currently two drugs available for SMA treatment. The first, Spinraza (nusinersen), was approved in 2016 with an antisense oligonucleotide (ASO) as an active substance [2,3]. It targets the dysfunctional gene SMN2 to synthesize more functional proteins responsible for the proper function of motor neurons. The second drug is Zolgensma used in gene therapy with adeno-associated viruses to deliver functional copies of the SMA-causing gene to supplement the defective gene [2,3]. 

Nusinersen is ordered into the cerebrospinal fluid and central nervous system tissue is the primary site of its action [4,5,6,7,8]. The active substance of a drug is cleared into the systemic circulation from cerebrospinal fluid [6,8]. The plasma concentrations of nusinersen remain relatively low compared to concentrations in the cerebrospinal fluid [6,7]. The plasma is the primary site for CSF clearance of this medicine. According to the literature, metabolism, and elimination of nusinersen occurs via exonuclease hydrolysis and urinary excretion [4,7]. The manufacturer suggests that the elimination of this drug is 135 to 177 days in the cerebrospinal fluid and 63 to 87 days in the plasma [7]. To date, however, little is known about their exact metabolites, or their concentrations in plasma or cerebrospinal fluid.

One of the first works in this area is Luu et al. [6] study. A hybridization nuclease-based enzyme-linked immunosorbent assay (ELISA) method, as well as the electrochemiluminescence method, were applied to determine OGN (nusinersen) in both cerebrospinal fluid and plasma. The sensitivities for both techniques were in the range of 0.05–1.5 ng/mL and were accurate to characterize the level of nusinersen in biological samples [6]. Although the methods appeared to be excellent for quantification of OGN, they can be characterized by one limitation—lack of metabolite determination and identification [6]. Another study presents the effects of nusinersen on the 1 H-NMR metabolomes of urine and serum of pediatric patients with SMA [9]. However whole metabolomes were analyzed and not specifically nusinersen metabolites. Results have shown that no significant differences were found in metabolomes before and after nusinersen therapy [9]. Kessler et al. [10] evaluated non-targeted cerebrospinal fluid proteomic profiles by mass spectrometry (MS) of patients treated with nusinersen. The different expressions of proteins related to neurodegeneration and neuroregeneration were noticed in patients with different types of SMA, and consequently may be suitable for diagnostic and predictive analyses [10]. They have applied MS for the analysis of proteins, but not for the study of nusinersen OGN active substance. This technique together with liquid chromatography (LC) is the most widely used for ASO investigations in biological samples [11,12,13,14]. The characterization and separation of OGNs by anion exchange chromatography, ion pair chromatography, and hydrophilic interaction liquid chromatography have been thoroughly reviewed. Nowadays increasing efforts are made to identify the impurities or metabolites of MS [11,12,13,14]. As the market for new medicinal, OGN-based drugs is growing, the analytical tools need to accelerate to provide higher precision, sensitivity, and resolution.

The purpose of this study was to develop a complex and reliable method for the analysis of serum obtained from SMA patients treated with nusinersen. An attempt was made to extract and separate metabolites of an OGN, which is the active substance of nusinersen. The resulting metabolites were identified at the same time with the use of mass spectrometry. To the best of our knowledge, this is the first publication presenting results of this kind.

## 2. Results and Discussion

### 2.1. Metabolites Identification

**Figure 1:**
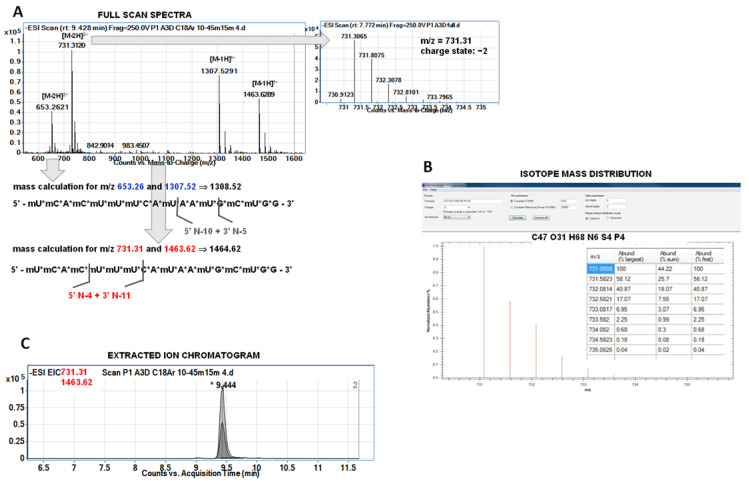
The scheme of nusinersen metabolites identification was performed during the present study: (**A**) full scan spectra with charge state determination for exemplary ion (used next for mass calculation); a scheme for the mass calculation of species at the spectra together with an assignment of metabolite; (**B**) isotope mass distribution for selected metabolite (1464.62 Da) used for additional confirmation based on the designation of ions which should appear at full scan spectra; (**C**) extracted ion chromatograms for pair of ions characteristic for one, exemplary metabolite, they were used for additional confirmation of metabolite identification since similar retention time for both ions is an indication that these signals come from a single compound. Notation: *—retention time for the two ions.

According to the Spinraza manufacturer and the literature data, ASO does not undergo oxidative metabolism [15,16,17]. One known exception is when OGN was administrated by inhalation and some oxidation products were identified in the lung sample [18]. However, the main metabolic pathway of nusinersen is sequential nucleotide deletion by 3′-exonucleases and 5′-endonucleases [2,4]. We did not have any standards for nusinersen metabolites, but we have attempted to identify metabolites based on the results of precise Q-TOF-MS analysis. The identification of metabolites was carried out in three different steps. First, it was attempted to identify which ions on the full scan spectrum come from a single compound. Each of the metabolites gave only two ions at full scan spectra. The charge state was assessed based on isotope distribution. Next, deconvolution was used for the calculation and determination of metabolite masses. Chromatograms for selected ions (EIC—extracted ion chromatogram) were then recorded. If the retention times for ions from a given metabolite were the same—this was considered as additional confirmation. Another identification confirmation was performed with the use of the isotope mass distribution tool used for metabolite mass calculation. For most of the metabolites, we have observed characteristic pairs of signals, the mass of one was double the mass of the other (e.g., the pair of signals *m*/*z* 617 Da and 1236 Da). These were signals from a single compound, which helped with identification. Figure 1 presents the scheme of identification performed during the study. Furthermore, an attempt was made to MS/MS fragmentation of selected metabolites ions. However, OGN fragmentation is not specific, which is similar to nusinersen metabolites. Regardless of the sequence and length, the same fragmentation ions were observed in the fragmentation spectra, which made it impossible to use them for additional identification. Fragment ions were: *m*/*z* = 126 for mU; *m*/*z =* 76 for methoxyethyl group from ribose modification, *m*/*z =* 96 for phosphorothioate group; *m*/*z =* 113 for ribose; *m*/*z =* 189 for ribose modified with methoxyethyl group; *m*/*z =* 285 for ribose with methoxyethyl and phosphorothioate group; *m*/*z =* 265 for guanosine, *m*/*z =* 240 for methyluridine.

### 2.2. The Influence of Amine in the Mobile Phase on the Signal’s Intensity and Peak Area

An important issue in metabolism studies is to obtain the highest possible sensitivity, which allows the detection of a greater number of metabolites. For this purpose, changes in the mobile phase composition can be made. In this study, we have used ion pair chromatography, which is the most common in the separation of OGNs. The mobile phases used in this mode of liquid chromatography are most often composed of HFIP and alkylamine. We tested three different alkylamines at a concentration of 5 mM. They were chosen based on our previous experience [19,20,21]. The amine type has a great influence on the separation of OGN mixtures. However, it also affects the ionization process in the electrospray ionization source, which was used in our study. On the other hand, effective ionization has a direct effect on the intensity of ion signals and peak areas [19,20,21]. 

The DIPEA, DMBA, and DPA were selected for the present study [19,21]. These amines provided the highest sensitivity for phosphorothioate, locked nucleic acids, 2’-O-methyl and 2’-O-methoxyethyl modified OGNs. Table 1 presents the peak areas at the EIC for eight selected ions of four metabolites. The lowest peak areas, and thus the lowest sensitivity, were observed for DPA. It was excluded from further studies as the amine that suppresses the signals of OGNs or does not allow their ionization as efficiently as DMBA and DIPEA. The peak areas for these two amines were comparable and may be used interchangeably, as the sensitivity for detecting OGN metabolites will be similar for both of them. Similar tendencies were observed for all other OGNs extracted from serum samples.

These effects are related to the type of amine and its properties. One of the parameters of importance in MS is the boiling point of amines, but it seems that it is not a critical parameter in the analysis of the nusinersen. DPA has a boiling point equal to 110 °C, DMBA 106 °C, and DIPEA 127 °C. The volatility of the alkylamines is not related to the peak areas of studied OGNs. The MS signal intensity depends also on the formation of gas-phase ions by ESI, which is related to the pH and pK_a_ values of amines in the mobile phase. The pK_a_ for each of the tested amines are very similar and range between 10 and 11. It is therefore not a parameter that differentiates the signal intensities of OGNs depending on the type of amine in the mobile phase. However, the structure of amines may be a parameter influencing the MS sensitivity of OGN analysis by ion pair chromatography [22]. DPA is a secondary amine, while DMBA and DIPEA are tertiary amines indicating that for secondary amines the sensitivity is lower [22]. According to Bartlett et al. [23], another parameter influencing the OGN signal intensity with regard to amine used in the mobile phase is Henry’s law constant. This parameter is the highest for DPA, while is low for DMBA (0.115 mol/m^3^ Pa) and DIPEA (0.065 mol/m^3^ Pa) [23]. The lower value of Henry’s law constant indicates sufficient volatility to evaporate from the droplet as OGNs are ionized. For this reason, it may be assumed that DMBA and DIPEA did not enrich the droplet and did not increase the alkylamine concentration, which would suppress ionization.

### 2.3. The Influence of MS Parameters on the OGN Signal Intensity

The following parameters of MS were optimized for the selection of conditions to obtain high signal intensities of OGNs: pressure in the nebulizer, capillary voltage, fragmentor, skimmer voltage, and skimmer. The EIC peak area and the signal intensities at the full scan spectra were compared. Tests were performed for two different mobile phases containing DMBA/HFIP, as well as for DIPEA/HFIP. Similar trends were observed in both cases. The low values of fragmentor voltage caused decreasing in EIC peak areas, similarly to the case of the skimmer and capillary voltage (Table S1). Contrary results were noticed for nebulizer gas pressure: high values decreased the intensities of OGN signals (Table S1). Finally, the peak area in the EIC was found to be maximum for a nebulizer pressure of 20 psi, capillary voltage of 4000 V, fragmentor voltage of 250 V, and a skimmer voltage of 100 V.

### 2.4. The Impact of Column and Stationary Phase on the Metabolite’s Separation

Since we did not have metabolite standards and metabolite identification is a complex process, we aimed to obtain the most complete separation of the compounds extracted from plasma. This greatly simplified identification, especially since OGNs are multiply charged compounds, as they tend to release hydrogen atoms from a phosphoric acid group and form negative ions. Consequently, the negative ions are present in different charge states at the spectra and multiple charge states complicate identification without standards. Therefore, correct and reliable identification without standards and at least partial separation is very difficult. 

Three different columns with different stationary phases were selected, namely octadecyl (C18), octadecyl with terminal phenyl group (C18-Ar), and octadecyl with an incorporated polar group (C18-AP). C18 column was chosen as the one most commonly used in OGN analysis, while C18 Ar was selected due to the presence of aryl packing, which should have better selectivity for OGNs (similar groups and double bonds are in nitrogenous bases). On the other hand, the C18 AP column was chosen because of its higher polarity compared to C18 and C18 Ar. 

**Figure 2:**
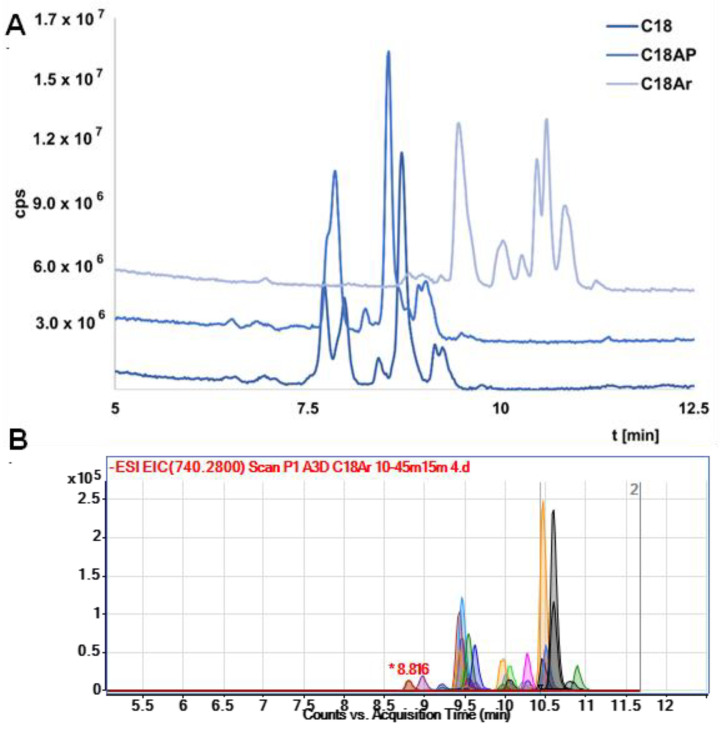
Exemplary separation results of metabolites in the extract obtained from one of the patients after administration of the third nusinersen dose: (**A**) TIC chromatograms for three different columns, (**B**) EIC for the C18Ar column. Experimental conditions: mobile phase composition 150 mM HFIP/5 mM DMBA, MeOH, gradient elution: 0 min–10% MeOH, 15 min–45% MeOH; mass spectrometric conditions: nebulizer gas pressure 20 psi, skimmer voltage 100 V, capillary voltage 4000 V, fragmentor voltage 250 V; for other conditions check Section 3.3 and Section 3.4. Notation: *—retention time for one ion.

It should be noted that in the initial stage of the study the resolution was low. Exemplary results were presented in Figure S1. Therefore, the gradient elution program was changed to increase the separation efficiency (Figure S2 presents exemplary results). The results (total ion chromatograms—TIC) for all columns obtained under the same chromatographic conditions are shown in Figure 2A. The lowest resolution was obtained for C18 AP, which is probably a consequence of hydrogen bonding between the OGNs and the polar group in the structure of the stationary phase. This stationary phase was excluded from further study. The results obtained for C18 and C18Ar differ mainly in retention—the OGNs were retained longer at the stationary phase with an additional aryl group (C18Ar). This effect is related to π…π interactions increasing the retention of tested compounds. The resolution for both columns is similar, although the elution order of the nusinersen metabolites is changed. However, both columns can be used to analyze serum extracts from patients who have received Spinraza. In Figure 2B the exemplary EIC chromatograms for metabolite separation with the use of C18 Ar are presented. Complete separation of all metabolites was not achieved, but it was sufficient enough to allow a more complete and reliable identification of studied compounds. For most metabolites, two ions were selected for which EIC was performed. Elution at the same time was an additional confirmation of the correct identification. Therefore, the best possible separation of metabolites was so important for the identification of compounds for which we did not have standards (nusinersen metabolites).

Furthermore, the coelution of several compounds at the same time (lack of separation) leads to suppression of ionization and the inability to fully identify some metabolites that are at low concentration levels or whose ionization is low.

Moreover, the use of a C18 Ar made it possible to identify, for example, two metabolites differing in mass by one (ion pairs: 696.76, 1394.52, and 697.25 and 1395.51) (Figure S3). In the case of the C18 and C18-AP, both compounds were eluted at the same time and initially mistakenly identified as one compound. For these reasons, the use of chromatographic columns that provide the most complete separation of compounds for which there are no standards is extremely important.

### 2.5. Serum Samples Preparation

In the next step of the study, we applied methods previously developed in our laboratory to prepare patient plasma samples for extraction of nusinersen and its metabolites. The protein removal was required for each sample. For this purpose, we chose the classical LLE method with a phenol(alkalized)/chloroform mixture. This method is used widely for many types of samples when efficient separation of proteins and nucleic acids is necessary. Another method is to use enzymatic degradation of proteins using proteinase K [18,24,25]. In our experience, both methods are equally effective. 

**Figure 3:**
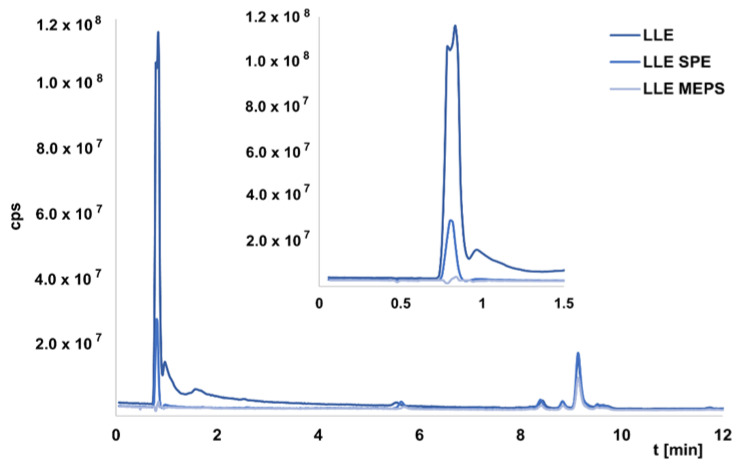
The TIC chromatograms for extracts from serum samples obtained with the use of LLE, LLE followed by SPE, and LLE followed by MEPS (for SDVB). Experimental conditions: Kinetex 1.7 µm EVO C18 column; mobile phase composition 150 mM HFIP/5 mM DMBA, MeOH; gradient elution: 10–45% *v*/*v* MeOH in 15 min; flow rate 0.3 mL/min; autosampler temperature 30 °C; column temperature 50 °C; injection volume 2.5 μL; mass spectrometric conditions: nebulizer gas pressure 20 psi, skimmer voltage 100 V, capillary voltage 4000 V, fragmentor voltage 250 V; drying gas flow 10 L/min; shielding gas flow 10 L/min; octopole voltage 800 V; drying gas temperature 350 °C; shielding gas temperature 400 °C.

In the next step, the LLE extracts were further purified and concentrated using other methods, namely SPE, MEPS, and MNPs. The extraction procedures were previously developed in our laboratory and used to extract OGNs from enriched serum samples [26,27,28]. In this study, we wanted to compare the efficiency of the developed methods in the study of real samples. It was not possible to determine exact recovery values because we did not have OGNs standards. Nevertheless, it was possible to compare the EIC areas between the chromatograms obtained for LLE extracts and the next method used. In the case of extracts obtained for LLE, samples injected into the chromatographic system were usually introduced into the MS after 4 min, so that any peaks originating from the matrix and eluted in the dead time would not cause suppression of ionization of the test compounds. Indeed, LLE extraction was insufficient to remove all substances from the matrix, resulting in their elution in the first minute of analysis. Figure 3 presents an exemplary chromatogram. The same figure shows example TIC chromatograms for selected extracts obtained after two-step LLE/SPE or LLE/MEPS, while Table 2 summarizes the recovery percentages (for five selected metabolites) for additional purification of LLE extracts. These were calculated as recoveries relative to the peaks areas values of the EIC for LLE extracts. The use of two-step extraction allowed for better purification of the sample compared to LLE. Each of the methods used, except for SPE, allowed the removal of compounds from the matrix that was present after LLE (Figure 3). The values given in Table 2 show that the recoveries of metabolites were the highest for SPE, but the standard deviation values were high, indicating that the method is not reproducible. The application of MNPs provided the lowest recovery with the highest standard deviation values (Table 2). The lower values were obtained for MEPS, but at the same time, complete purification of the sample was possible (Figure 3). Comparing two different adsorbents used for extraction revealed that the application of SDVB provides greater recoveries (Table 2).

We believe that SPE is a good method for plasma sample purification, but MEPS appears to be the most suitable method because despite lower recovery values, the reproducibility is high and the purification is very efficient.

### 2.6. Metabolites Identified in Serum Extracts

**Figure 4:**
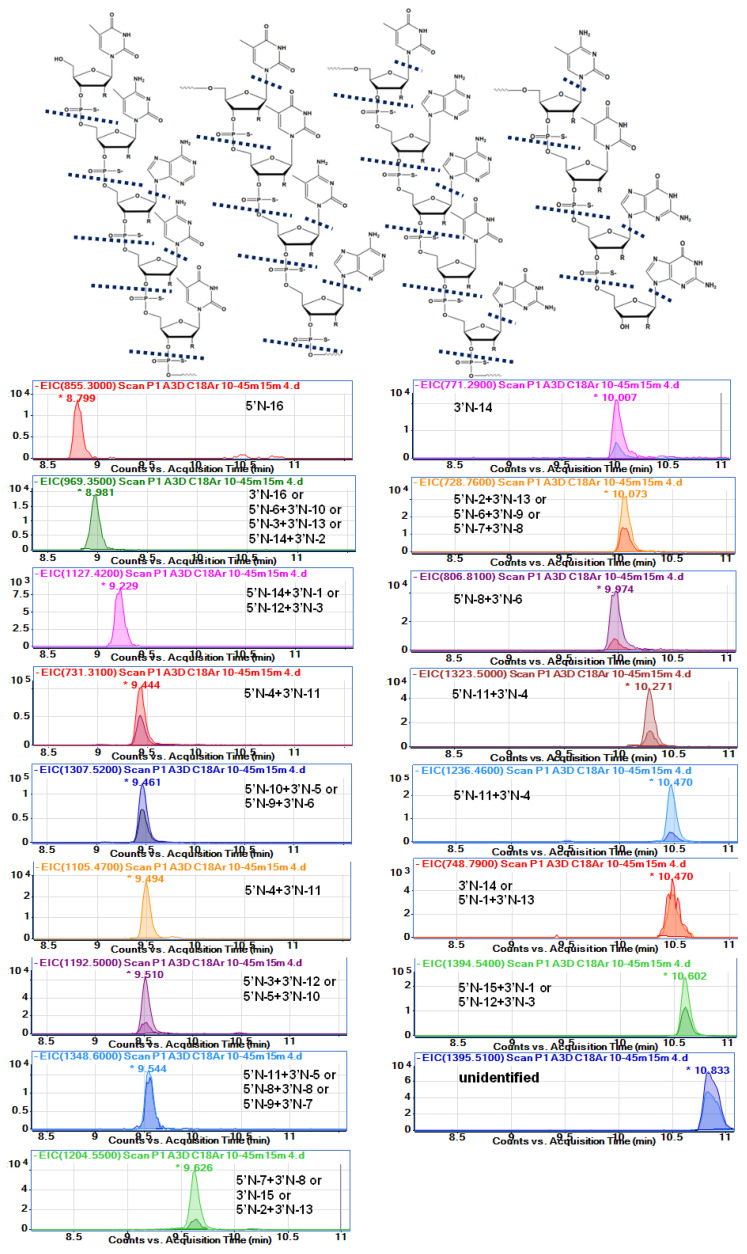
The EIC chromatograms with identified metabolites for an extract from serum sample obtained with the use of LLE followed by MEPS. Experimental conditions: ACE C18 Ar 1.7 µm column; mobile phase composition 150 mM HFIP/5 mM DMBA, MeOH; gradient elution: 10–45% *v*/*v* MeOH in 15 min; flow rate 0.3 mL/min; autosampler temperature 30 °C; column temperature 50 °C; injection volume 2.5 μL; mass spectrometric conditions: nebulizer gas pressure 20 psi, skimmer voltage 100 V, capillary voltage 4000 V, fragmentor voltage 250 V; drying gas flow 10 L/min; shielding gas flow 10 l/min; octopole voltage 800 V; drying gas temperature 350 °C; shielding gas temperature 400 °C. *—retention time for the two ions.

Analysis of therapeutic OGNs faces many challenges, especially during metabolite identification, due to the mass spectral complexity of larger macromolecules. They produce several charge states, which may overlap signals of metabolites commonly generated by sequential removal of nucleotides from the full-length OGN. Nusinersen metabolites are not quite such an example: their ionization is relatively low, which does not cause a large number of signals from different charge states. Despite this, however, the identification was not an easy task, as it required a very careful assignment of *m*/z and masses values to unambiguously identify metabolites. Table 3 and Figure 4 show the probable identification results obtained for serum extract collected from a person administrated with Spinraza. The table shows *m*/*z* values observed in the full scan spectra (after compounds separation) and *m*/*z* values obtained from isotope mass distribution. Moreover, the retention times at EIC were provided, together with calculated molecular masses, molecular formulas, and shortcuts for metabolites. Figure 4 presents the EIC chromatograms for various metabolites, as well as the nusinersen structure with indicated probable cleavage places. To the best of our knowledge, this is the first attempt to fully identify the metabolites of nusinersen.

Despite the well-known fact that phosphorothioate and 2′-*O*-methoxyethyl modifications decrease nuclease-mediated degradation, nusinersen was metabolized [15,18,19,29,30]. The 3′end shortmers and 5′end shortmers were generated and identified in serum. The results suggest that both 3′ exonucleases and 5′ exonucleases were responsible for metabolizing nusinersen in the human body. On the other hand, depurination and depyrimidination were noticed with simple loss of bases (Table 3). Fifteen different probable metabolites were identified. Characteristically, only metabolites with relatively low masses compared to nusinersen were observed. The range of masses observed for the degradation products was between 855 and 1614 Da, while the mass of nusinersen is over 7000 Da. Consequently, the metabolites were 2-mer, 3-mer, or 4-mer oligonucleotides.

The sequence of nusinersen is not very specific if we consider such short fragments of this compound. In the case of, for example, a metabolite that is a dinucleotide with a G*G* sequence, there is only one such fragment in the whole nusinersen molecule. For this reason, there is no problem with precise metabolite identification (5′N-16). In contrast, in the case of the metabolite mU*mC*, there are 4 such repeats in the entire nusinersen sequence, and there may be four metabolites formed by degradation from both the 3’ and 5’ ends. All of them will have exactly the same masses, and it is therefore impossible to determine exactly which metabolite it is. A similar situation applies to metabolites build up of three nucleotides, e.g., for a mass of 1205 Da the metabolite will be either a fragment with the sequence mC*A*mU* (5’N-7+3’N-8) or with the sequence mU*mC*A*, for which there are two metabolite possibilities: 3’N-16 and 5’N-6+3’N-9. For these reasons, accurate and clear identification of metabolites was not possible in this work. Table 3 summarises the masses of the metabolites with all possible sequences for each of them. 

The nusinersen was not observed in any of the analyzed extracts (Figure 4, Table 3). There could be three reasons for this effect: insufficient elution strength of the final mobile phase composition during the gradient (too little MeOH content); low concentration of nusinersen in serum; total metabolism of this compound. The first reason was excluded during the study as different gradients were used and even with 80% MeOH in the mobile phase, no other OGNs were eluted from the column (apart from the ones identified in this study). The other two possibilities in our opinion are equally possible. Apparently, nusinersen is practically completely enzymatically degraded to its metabolites in tested serum samples. Although this contradicts the data provided by the drug manufacturer, who declares that nusinersen was present in the plasma of patients who were administered Spinraza [2,3]. 

Only two metabolites can be identified as only 5’shortmer or 3’shortmer (Table 3, Figure 4). Most of the identified metabolites are products of exonuclease (3’- and 5’)-mediated hydrolysis simultaneously at both ends of the sequence (for both 3’ and 5’). Thirteen such metabolites have been identified. As they are mainly composed of three nucleotides, the sequences of which are not sufficiently specific, it is difficult to indicate undoubtedly whether enzymatic hydrolysis took place to a greater extent at the 3’ or 5’ end. An additional loss of nitrogenous bases was observed for some metabolites (970 Da, 1464 Da, 1349 Da, 1459 Da) (Table 3, Figure 4). These data are to some extent in accordance with Spinraza manufacturer; however, they claim that N-1 metabolites of the drug are present in the cerebrospinal fluid, while N-1,2,3 metabolites were predominantly identified in the plasma [2,3,4]. Our results do not entirely support these conclusions, as we also observed depurination and depyrimidination. 

The most difficult identification problems concerned two compounds that were initially identified as one. These were compounds with *m*/*z* equal to 696.76 and 1394.54, as well as 697.25 and 1395.51. Both compounds were fully separated (Figure S2B), clearly indicating that they are two different metabolites. The former should have a mass of 1395.41 Da and the latter 1369.5 Da (Table 3). The first compound is a product of 3’- and 5’exonuclease mediated hydrolysis together with depurination. In the case of a metabolite of 1369.5 Da, it is difficult to identify its exact sequence. Perhaps it is a sequence isomer of a metabolite with a mass of 1 Da less or maybe this metabolite is a different compound. However, without standards of both metabolites, their identification is very difficult, and therefore, their identification was not unequivocally confirmed in this study. 

### 2.7. Changes in Metabolites Level in Serum Samples

We will present the sample analysis results obtained for two patients in this research. They are primarily intended to demonstrate the utility of the methods developed during this research. In the case of the second patient (P2), samples were taken at the beginning of the COVID-19 pandemic when patients stayed in the hospital for a very short time, so results were obtained for samples taken before the drug was administered (before the first BID and second BIID dose) (children were sent home after administration) (Table 4).

Table 4 presents the results. Most metabolites were identified in both P1 and P2 plasma, but it should be noted that their concentration was usually lower in P2 plasma (smaller area per EIC) (except for metabolites of following masses: 1544 Da, 1459 Da, 1324 Da 1237 Da). The amount of nusinersen in P2 plasma was also lower compared to P1. These results may suggest a different metabolism of the drug depending on the patient’s organism, but there are still too few results and studies need to be extended. The metabolites of masses 970 Da, and 1349 Da were not detected in P2 plasma extracts, but this may be due to the very low concentration of these compounds in plasma (below the detection limit of the developed LC-MS method) (Table 4).

Plasma samples were collected before and 24 h after drug administration. An interesting result is that for P1 there were no metabolites in the plasma 24 h after drug administration of P1 AID (Table 4). The opposite effect was observed for P2–P2 AID. These effects may suggest a different distribution of the drug in the body, but more precise studies on a larger group of patients are needed. 

When comparing the differences in the number of metabolites before and after administration of the drug for P1, it can be observed that they increase after administration of the drug, which is an understandable effect (Table 4). The highest amount of metabolites was found in plasma before and after the third dose, and they decreased with the fourth dose. This is probably due to the long interval between the third and fourth dose (35 days). However, it should be emphasized that plasma is saturated with the drug’s metabolites throughout the time it is administered to children. The amount of nusinersen in the plasma was less than the number of metabolites (Table 4). It can therefore be concluded that the compound reaches the plasma mainly in a metabolized form.

## 3. Materials and Methods

### 3.1. Materials

Nusinersen is the sodium salt of an 18-mer 2’-O-methyl-phosphorothioate oligoribonucleotide dissolved in sodium dihydrogen phosphate dihydrate, disodium phosphate, sodium chloride, potassium chloride, calcium chloride dihydrate, magnesium chloride hexahydrate, sodium hydroxide, hydrochloric acid, and water (Spinraza™). The full sequence of the ASO is 3’-mU-mC-A-mC-mU-mU-mU-mC-A-mU-A-A-mU-G-mC-mU-G-G-5′, while the molecular mass equals 7110 g/mol.

Mobile phases were prepared using high purity solvents such as methanol, 1,1,1,3,3,3-hexafluoro-2-propanol (HFIP), as well as *N*,*N*-dimethylbutylamine (DMBA), *N*,*N*-diisopropylethylamine (DIPEA), dipropylamine (DPA) and LC-MS water (Merck KGaA, Darmstadt, Germany). The mixture of phenol/chloroform/isoamyl alcohol (25:24:1) (VWR International, Gdańsk, Poland), acetonitrile, chloroform, 10 mM ammonium acetate (pH 4.5 and 9.0), acetic acid, ammonia solution 25% (Sigma-Aldrich, Gillingham, Dorset, UK) was also used during sample preparation step.

### 3.2. Instrumentation

The 1260 Infinity Quaternary System (Agilent, Waldbronn, Germany) ultra-high-performance liquid chromatography (UHPLC) system with a binary pump, vacuum chambered microdegasser, thermostatically controlled autosampler, column compartment, and a diode-array detector (DAD) was applied. The system was equipped with Agilent 6540 UHD Accurate-Mass Quadrupole Time-of-Flight (Q-TOF) mass spectrometer (Waldbronn, Germany). Electrospray ionization was used in the negative ion mode and full scan mass spectra were recorded within the mass range of *m*/*z* 450–1700. The data were collected with the use of Agilent Mass Hunter Software (version B.04.01). 

Microextraction by packed sorbents (MEPS) was performed using a 100-μL volume eVol XR digital analytical syringe (Trajan Scientific, Ringwood, Victoria, Australia). The MEPS Barrel Insert and Needle Assemblies (BINs) were purchased from MS Spektrum (Warsaw, Poland). Two different adsorbents were used: poly(styrene-co-divinylbenzene) (SDVB) and octadecyl (C18). Each BIN contained ~4 mg of the sorbent with a particle size of 45 µm. The Baker SPE 12-G (J.T.Baker, Deventer, Holland) chamber was also utilized during the study. Oasis^®^ HLB (30 mg, 1 mL) (Waters, MA, USA) cartridge was used. Samples were concentrated with the use of a CentriVap vacuum concentrator (Labconco, Kansas City, MO, USA), while they were centrifuged by a 5424 microliter Eppendorf AG centrifuge (Hamburg, Germany). 

### 3.3. MS Parameters Optimization

Optimization of Q-TOF-MS was performed for several parameters: nebulizer gas pressure (10–60 psi), skimmer voltage (40–100 V), capillary voltage (3000–4000 V), and fragmentor voltage (100–250 V). The area of peaks on EIC chromatograms was measured and compared. All of these parameters were changed for similar chromatographic conditions: 5–80 % *v*/*v* MeOH in 15 min for 5 mM DMBA/150 mM HFIP; flow rate 0.3 mL/min; 1 μL injection; autosampler temperature 30 °C, column temperature 50 °C, column Kinetex 1.7 µm EVO C18 (100 × 2.1 mm) (Phenomenex, Torrance, CA, US). The other Q-TOF-MS parameters, such as drying gas flow (10 L/min), shielding gas flow (10 L/min), octopole voltage (800 V), drying gas temperature (350 °C), and shielding gas temperature (400 °C) were selected based on our previous experience with antisense OGNs studies using Q-TOF-MS [29,30]. 

### 3.4. Chromatographic Conditions

OGN metabolite separation was performed with the use of ion-pair chromatography. Three different columns were tested for separation selectivity: ACE C18-Ar, ACE Excel C18-AP (1.7 μm; 2.1 × 100 mm) (Advanced Chromatography Technologies, Aberdeen, Scotland), and Kinetex 1.7 µm EVO C18 (2.1 × 100 mm) (Phenomenex, Torrance, CA, USA). The mobile phase flow rate was 0.3 mL/min. The autosampler temperature was 30 °C, while the column was kept at 50 °C. The injection volume was 2.5 μL. The mobile phases tested during the study contained methanol, HFIP, as well as DMBA or DIPEA, or DPA. The gradient elution was applied (gradient details were provided in the text and figure captions).

### 3.5. Sampling

A total of 2 pediatric patients (P1 and P2) with genetically confirmed SMA 2 or 3 were enrolled at the Division of Developmental Neurology, the Medical University of Gdansk at the University Clinical Centre from January 2020 to May 2020. Each clinical indication to start treatment on nusinersen was provided by neurologists with longstanding experience in neuromuscular diseases. The serum samples were taken before intrathecal administration of nusinersen (12 mg dissolved in 5 mL of solution) on treatment days 0 (before first dose BID, after first dose AID), 14 (before second dose BIID, after second dose AIID), 28 (before third dose BIIID, after third dose AIIID), and 63 (before fourth dose BIVD, after fourth dose AIVD) of the loading dosing. Samples were taken by relevant guidelines of Ludwik Rydgier Collegium Medicum of Nicolaus Copernicus University in Toruń and approved by the Bioethical Commission of the Nicolaus Copernicus University (permission no. 707/2019). Serum samples were immediately frozen and after thawing they were prepared by the methods described in Section 3.6.

### 3.6. Sample Preparation

The serum samples were diluted with redistilled water at a ratio of 1:3. Next, the liquid–liquid extraction (LLE) with the use of phenol/chloroform/isoamyl alcohol mixture (25:24:1 *v*/*v*) was applied. The 3 mL of phenol/chloroform/isoamyl alcohol mixture was added to the 3 mL of diluted serum sample and mixed. Next, the suspension was centrifuged (RCF = 20,160× *g* for 20 min). The supernatant was transferred to another tube and then an additional LLE extraction step was made with the use of chloroform (at a ratio of 1:4). These additional extractions were repeated three times to remove phenol residues. 

We also used three other procedures for additional sample purification, which we applied directly to extracts after LLE using phenol/chloroform/isoamyl alcohol (before extraction with chloroform). These procedures were developed previously in our laboratory for the extraction of antisense OGNs from plasma samples [26,27,28].

Solid-phase extraction (SPE) [26]:

Adsorbent: Oasis HLB (30 mg, 1 mL) (Waters, MA, USA).

Conditioning: 1 mL acetonitrile, 1 mL water, 1 mL 5 mM DMBA/150 mM HFIP (left for 30 min).

Sample load: 100 µL of LLE extract + 100 µL IPR 5 mM DMBA/150 mM HFIP (left for 30 min before load).

Washing: 400 µL 90% *v*/*v* 5 mM DMBA/150 mM HFIP, 10% *v*/*v* MeOH.

Elution: 400 µL 10% *v*/*v* 5 mM DMBA/150 mM HFIP, 90% *v*/*v* MeOH.

Remark: samples were evaporated to dryness and residue was dissolved in 100 µL of 5 mM DMBA/150 mM HFIP.

Microextraction by packed sorbent (MEPS) [27]:

Adsorbents: poly(styrene-co-divinylbenzene) (SDVB) and octadecyl (C18).

Conditioning: 100 µL methanol, 100 µL water, 5 × 50 µL DMBA/150 mM HFIP. 

Sample load: 50 µL of LLE extract + 50 µL IPR 5 mM DMBA/150 mM HFIP (5 x draw/eject).

Washing: 100 µL 90% *v*/*v* 5 mM DMBA/150 mM HFIP, 10% *v*/*v* MeOH.

Elution: 100 µL 10% *v*/*v* 5 mM DMBA/150 mM HFIP, 90% *v*/*v* MeOH (2 × draw/eject).

Remark: flow 100 µL/min; samples were evaporated to dryness and residue was dissolved in 50 µL of 5 mM DMBA/150 mM HFIP.

Magnetic nanoparticles (MNPs) [28]:

Adsorbent: 2 mg of zwitterionic, imidazolium-based crosslinked poly(ionic liquid) with acetic group incorporated in imidazole ring (check Supplementary materials for details).

Conditioning: 100 µL MeOH, 100 µL 10 mM ammonium acetate pH = 4.5.

Sample load: 33 µL LLE extract + 67 µL 10 mM ammonium acetate pH = 4.5.

Washing: 100 µL 10 mM ammonium acetate pH = 4.5.

Elution: 50 µL 10 mM ammonium acetate pH = 9.5/MeOH 50/50 *v*/*v* (30 min mixing).

Remarks: each time magnetic separation was performed.

## 4. Conclusions

The present study resulted in the identification of metabolites of the active substance of Spinraza, which is used to treat SMA. Several factors had a major impact on the quality of the results obtained and the reliability of the identification. In particular, the composition of the mobile phase is crucial for the intensity of the signals. It was also influenced by the correct choice of operating parameters of the mass spectrometer. The selection of the proper stationary phase turned out to be crucial for the separation of the metabolites. Without separation, the spectra obtained were very complex, making the identification of compounds difficult, or even impossible, or erroneous (e.g., erroneous identification of ions with similar masses as one compound). According to the authors, this is one of the most important achievements of this work—a demonstration of how important separation is in the identification of OGN metabolites. Moreover, the additional sample purification experiments also showed that plasma samples should be purified after the initial removal of proteins. Two of the methods used gave satisfactory results in terms of recovery. Both used solid-phase extraction; however, solid-phase microextraction provided better reproducibility of results, with less sample and other solvents used. The careful development of sample preparation, OGN separation, and determination methods allowed for successful metabolite studies.

The major metabolic pathway of nusinersen involves the formation of 3’ and 5’ shortmers. The exonuclease (3’- and 5’)-mediated hydrolysis led also to the nucleotide cleavage simultaneously at both ends of the nusinersen sequence. Furthermore, depurination and depyrimidination were also involved in the metabolism, as nucleobase losses were observed during the study. Differences in the levels and amounts of metabolites were observed between patient plasma, which may suggest differences in the metabolism of Spinraza depending on the child’s body. Some metabolites were detected only in the plasma of one of the patients. However, the results that we have obtained in this study are still too few to draw general and definite conclusions. However, one regularity was observed—the amount of metabolites 24 h after administration of drugs increased, which was an expected effect. 

The research certainly needs to be continued and expanded. This will now be possible because a very good method has been developed in the present study, which allows for OGN extraction, purification, more complete separation, and identification. Using this method, the research can be successfully continued.

## Figures and Tables

**Table 1:** The peak areas determined for various ions at extracted ion chromatograms (EIC) for extract obtained from one of the patients after administration of the third nusinersen dose. Experimental conditions: Kinetex 1.7 µm EVO C18 column, mobile phase: 150 mM HFIP/5 mM amine, MeOH, gradient elution: 0 min–10% MeOH, 15 min–45% MeOH; mass spectrometric conditions: nebulizer gas pressure 20 psi, skimmer voltage 100 V, capillary voltage 4000 V, fragmentor voltage 250 V; for other conditions check Section 3.3 and Section 3.4.

Ion	Metabolite	Peak area at EIC		
		DIPEA	DMBA	DPA
731.31	5′N-4+3′N-11	628,828 ± 1381	599,944 ± 1141	410,260 ± 1742
1463.62		256,784 ± 1997	266,642 ± 895	166,768 ± 1062
806.82	5′N-8+3′N-6	225,819 ± 869	232,919 ± 708	195,687 ± 891
1614.61		49,786 ± 902	50,006 ± 554	37,733 ± 399
696.76	5′N-15+3′N-1	447,757 ± 1853	492,409 ± 1021	341,113 ± 1660
1394.54		965,600 ± 1003	970,155 ± 970	829,201 ± 1236
661.29	5′N-11+3′N-4	25,897 ± 651	30,844 ± 222	12,599 ± 274
1323.58		43,497 ± 745	52,681 ±386	35,664± 186

**Table 2:** The recovery values of selected metabolites from LLE extracts for different methods.

Metabolite	Ion	Recovery of Metabolites from LLE Extracts [%]			
		SPE	MEPS with SDVB	MEPS with C18	MNPs
5′N-10+3′N-5 or 5′N-9+3′N-6	653.26	91 ± 2	85 ± 1	78 ± 2	66 ± 5
	1307.52	92 ± 1	87 ± 2	80 ± 1	63 ± 2
5′N-11+3′N-4	661.24	93 ± 3	84 ± 2	77 ± 1	66 ± 3
	1323.50	94 ± 4	84 ± 2	76 ± 1	63 ± 4
5′N-11+3′N-4	617.73	98 ± 3	86 ± 1	80 ± 2	65 ± 4
	1236.46	97 ± 4	83 ± 2	78 ± 3	65 ± 4
5′N-15+3′N-1 or 5′N-12+3′N-3	696.76	96 ± 4	80 ± 1	79 ± 1	63 ± 5
	1394.54	96 ± 3	81 ± 1	78 ± 1	68 ± 3
3′N-14	771.29	90 ± 4	86 ± 1	76 ± 2	71 ± 4
	1543.59	91 ± 5	86 ± 2	75 ± 2	69 ± 3

Where: LLE: liquid-liquid extraction; MEPS—microextraction by packed sorbent, SDVB—poly(styrene-co-divinylbenzene; MNPs—magnetic nanoparticles.

**Table 3:** The *m*/*z* values for observed ions, their charge states, retention times, and identified metabolites.

Observed Ions [*m*/*z*]	Charge State	Retention Time [min]	Ions at Isotope Mass Distribution [*m*/*z*]	Metabolite			
				Deconvoluted Mass [Da]	Predicted Mass [Da]	Sequence	Probable METABOLITES
855.30	−1	8.81	855.30	856.30	855	G*G*	5′N-16
969.35	−1	8.98	969.92	970.35	971	mU*mC*MoeR mC*mU*MoeR	3′N-16 or 5′N-6+3′N-10 or 5′N-3+3′N-13 or 5′N-14+3′N-2
1127.42	−1	9.21	1127.35	1128.42	1128	G*mU*mC mU*G*mC	5′N-14+3′N-1 or 5′N-12+3′N-3
731.31	−2	9.44	731.35	1464.62	1464	*mU*mU*mU*MoeR	5′N-4+3′N-11
1463.62	−1		1463.73				
653.26	−2	9.46	653.21	1308.52	1308	*A*A*mU* *mU*A*A*	5′N-10+3′N-5 or 5′N-9+3′N-6
1307.52	−1		1307.43				
1105.47	−1	9.48	1105.37	1106.47	1104	mU*mU*mU	5′N-4+3′N-11
595.74	−2	9.51	593.48	1193.49	1194	mC*mU*mU* mU*mU*mC*	5′N-3+3′N-12 or 5′N-5+3′N-10
1192.50	−1		1185.97				
673.80	−2	9.54	673.84	1349.6	1349	MoeR*mU*A*MoeR* MoeR*A*mU*MoeR*	5′N-11+3′N-5 or 5′N-8+3′N-8 or 5′N-9+3′N-7
1348.60	−1		1348.70				
601.77	−2	9.63	602.07	1205.55	1205	mC*A*mU* mU*mC*A* A*mC*mU*	5′N-7+3′N-8 or 3′N-15 or 5′N-2+3′N-13
1204.55	−1		1205.15				
806.81	−2	9.97	806.85	1615.62	1614	A*mU*A*A*	5′N-8+3′N-6
1614.62	−1		1614.71				
771.29	−2	10.00	771.23	1544.59	1543	G*G*mU*mC	3′N-14
1543.59	−1		1543.48				
728.76	−2	10.04	728.72	1459.52	1458	A*mC*mU*MoeR* mU*mC*A*MoeR* mC*A*mU*MoeR*	5′N-2+3′N-13 or 5′N-6+3′N-9 or 5′N-7+3′N-8
1458.52	−1		1458.45				
661.24	−2	10.28	661.29	1324.49	1325	*A*mU*G*	5′N-11+3′N-4
1323.50	−1		1323.58				
617.73	−2	10.45	617.59	1237.46	1233	A*mU*G*	5′N-11+3′N-4
1236.46	−1		1236.39				
748.79	−2	10.45	748.73	1499.47	1499	mU*mC*A*mC	3′N-14 or 5′N-1+3′N-13
1498.58	−1		1498.47				
696.76	−2	10.60	696.70	1395.41	1395	G*mU*mC*MoeR mU*G*mC*MoeR	5′N-15+3′N-1 or 5′N-12+3′N-3
1394.54	−1		1394.41				
697.25	−2	10.83	697.30	1396.5	1396	unidentified	—
1395.51	−1		1395.41				

Where: *—phosphorothioate group; More—2′-O-methoxyethylribose.

**Table 4:** The peak areas at EIC for different metabolites and two patients (P1 and P2) administrated with nusinersen.

Observed Ions [*m*/*z*]	Metabolite	P1								P2		
		BID	AID	BIID	AIID	BIIID	AIIID	BIVD	AIVD	BID	AID	BIID
855.30	5′N-16	−	−	22,292	41,846	60,277	91,142	84,509	99,599	−	27,448	12,001
969.35	3′N-16 or 5′N-6+3′N-10 or 5′N-3+3′N-13 or 5′N-14+3′N-2	−	−	60,944	87,659	100,005	138,235	98,860	107,452	−	35,669	19,241
1127.42	5′N-14+3′N-1 or 5′N-12+3′N-3	−	−	20,356	47,092	28,043	65,380	18,313	40,026	−	−	−
731.31	5′N-4+3′N-11	−	−	375,922	684,992	235,724	889,988	367,031	320,948	−	111,877	81,756
1463.62		−	−	386,093	709,428	288,171	923,407	189,957	976,055	−	30,627	19,004
653.26	5′N-10+3′N-5 or 5′N-9+3′N-6	−	−	479,340	549,774	509,698	650,947	484,355	513,908	−	100,994	61,892
1307.52		−	−	439,999	551,092	524,610	5,416	371,283	499,875	−	92,650	55,528
1105.47	5′N-4+3′N-11	−	−	38,654	49,387	90,130	143,078	113,883	438,601	−	30,845	20,516
595.74	5′N-3+3′N-12 or 5′N-5+3′N-10	−	−	43,878	76,738	83,663	129,864	100,645	123,984	−	17,933	11,278
1192.50		−	−	117,383	183,000	214,185	318,363	224,654	270,008	−	46,995	32,846
673.80	5′N-11+3′N-5 or 5′N-8+3′N-8 or 5′N-9+3′N-7	−	−	19,746	24,874	20,503	45,787	16,843	24,777	−	−	−
1348.60		−	−	26,803	35,091	37,870	96,701	38,993	70,664	−	−	−
601.77	5′N-7+3′N-8 or 3′N-15 or 5′N-2+3′N-13	−	−	78,121	96,364	114,024	149,239	98,453	116,630	−	26,008	15,541
1204.55		−	−	290,662	370,111	407,015	617,843	296,511	454,855	−	51,278	27,922
771.29	3′N-14	−	−	119,743	150,487	161,380	192,834	193,251	235,326	−	250,180	257,313
1543.59		−	−	22,891	29,055	30,772	37,718	51,847	42,685	−	51,660	51,872
728.76	5′N-2+3′N-13 or 5′N-6+3′N-9 or 5′N-7+3′N-8	−	−	99,638	134,029	163,227	241,621	276,904	318,706	−	190,819	164,560
1458.52		−	−	35,011	49,366	53,134	79,228	91,123	97,483	−	68,820	55,182
806.81	5′N-8+3′N-6	−	−	246,981	350,000	408,377	711,294	316,112	340,207	−	219,256	169,060
1614.62		−	−	107,999	163,227	218,298	116,891	46,764	57,868	−	33,602	23,482
661.24	5′N-11+3′N-4	−	−	91,447	120,778	129,391	145,323	167,028	166,360	−	140,036	108,420
1323.50		−	−	156,883	200,827	264,920	287,088	253,539	284,406	−	270,916	231,482
617.73	5′N-11+3′N-4	−	−	141,226	196,889	188,981	445,875	441,159	707,505	−	636,703	547,665
1236.46		−	−	504,634	730,087	634,256	1,365,200	1,488,668	1,749,378	−	2,076,167	1,958,401
748.79	3′N-14 or 5′N-1+3′N-13	−	−	10,007	14,763	16,877	24,332	19,870	21,998	−	9643	12,855
1498.58		−	−	13,928	18,995	26,743	36,909	31,651	34,037	−	10,273	14,334
696.76	5′N-15+3′N-1 or 5′N-12+3′N-3	−	−	640,294	911,653	1,149,054	1,203,453	903,786	1,063,517	−	202,241	184,736
1394.54		−	−	776,380	1,069,312	1,382,465	1,590,716	900,278	1,292,842	−	242,374	227,235
697.25	unidentified	−	−	369,944	523,095	634,515	899,517	388,974	483,546	−	269,885	176,447
1395.51		−	−	285,092	409,661	454,274	711,694	232,607	357,814	−	226,691	132,025

Where: BID: before first dose, AID: after first dose AID, BIID: before second dose, AIID: after second dose, BIIID: before third dose, AIIID: after third dose, BIVD: before fourth dose, AIVD: after fourth dose.

## Data Availability

Not applicable.

## Supplementary Materials

The following supporting information can be downloaded at: https://www.mdpi.com/article/10.3390/ijms231710166/s1.
