# Supplementary material for: Development of the Method for Nusinersen and Its Metabolites Identification in the Serum Samples of Children Treated with Spinraza for Spinal Muscular Atrophy
Source: Int J Mol Sci. 2022 Sep 5;23(17):10166. doi: 10.3390/ijms231710166 (PMC9456300; doi:10.3390/ijms231710166)
Supplement: Supplementary file 1 [file ijms-23-10166-s001.zip › ijms-1860396-supplementary.pdf]

Development of the method for nusinersen and its metabolites identification in the serum samples of children treated with Spinraza for spinal muscular atrophy

Sylwia Studzińska[a,*], Maria Mazurkiewicz-Bełdzińska[b], Bogusław Buszewski[a]

[a] Chair of Environmental Chemistry and Bioanalytics, Faculty of Chemistry, Nicolaus Copernicus University in Toruń, 7 Gagarin Str., PL-87-100 Toruń (Poland),
tel. (48)(56)6114308, fax. (48)(56)6114837,
[b] Department of Developmental Neurology, Medical University of Gdansk, 7 Dębinki Str., PL-80-952, Gdańsk, Poland
*kowalska@chem.umk.pl

The procedure for the synthesis of magnetic nanoparticles (MNPs) coated with zwitterionic, imidazolium-based crosslinked poly(ionic liquid) (with acetic group incorporated in imidaloze ring) [20]

The polymerizable ILs with a vinyl functional group was used together with the Fe_3O_4 particles. Their surface was firstly modified with 3-(methacryloxy)propyltrimethoxysilane (MPS). Next, Fe_3O_4-MPS were coated with poly(ionic liquid) crosslinked with divinylbenzene. The coating was performed by miniemulsion polymerization of the synthesized ILs in the presence of DVB as a cross-linker and $K_2S_2O_8$ as an initiator. Coating of MNPs with zwitterionic, crosslinked poly(ionic liquid)s was performed in two steps: 1. the coating of Fe_3O_4 with the polymer with protected carboxyl groups (in form of ethyl ester); 2. deprotectection by the basic hydrolysis [20]. The structure of MNPs used in the study is presented below.

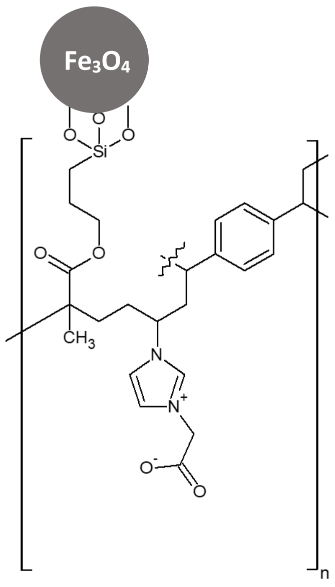

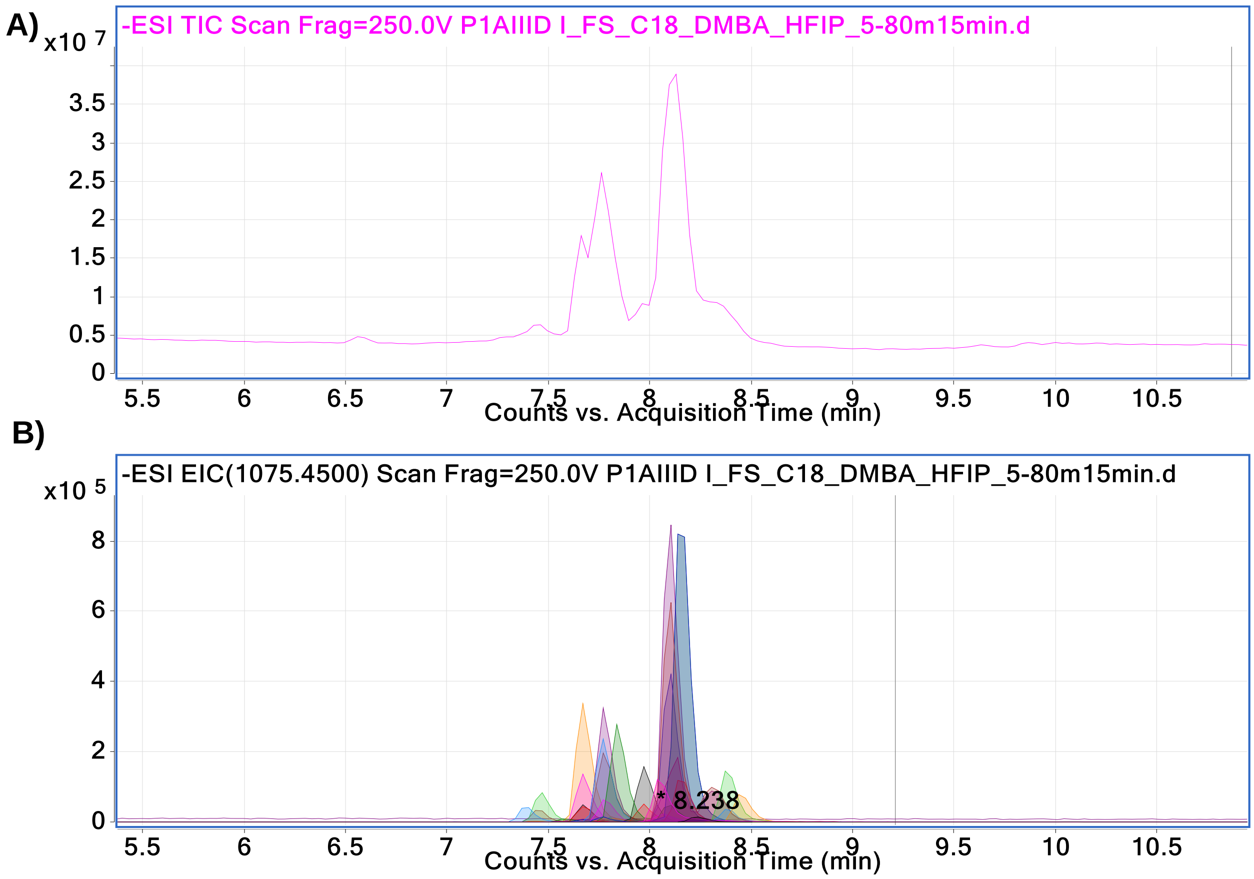

Figure S1. Exemplary separation results of metabolites in the extract obtained from one of the patients after administration of the third nusinersen dose: A) TIC, B) EIC. Experimental conditions: Kinetex 1.7µm EVO C18 column; mobile phase composition 150mM HFIP/5mM DMBA, MeOH; gradient elution: 5-80% MeOH in 15 minutes; flow rate 0.3 ml/min; autosampler temperature 30 °C, column temperature 50 °C; injection volume 2.5 µL; mass spectrometric conditions: nebulizer gas pressure 20psi, skimmer voltage 100V, capillary voltage 4000V, fragmentor voltage 250V; drying gas flow 10 l/min; shielding gas flow 10 l/min; octopole voltage 800V; drying gas temperature 350°C; shielding gas temperature 400 °C.

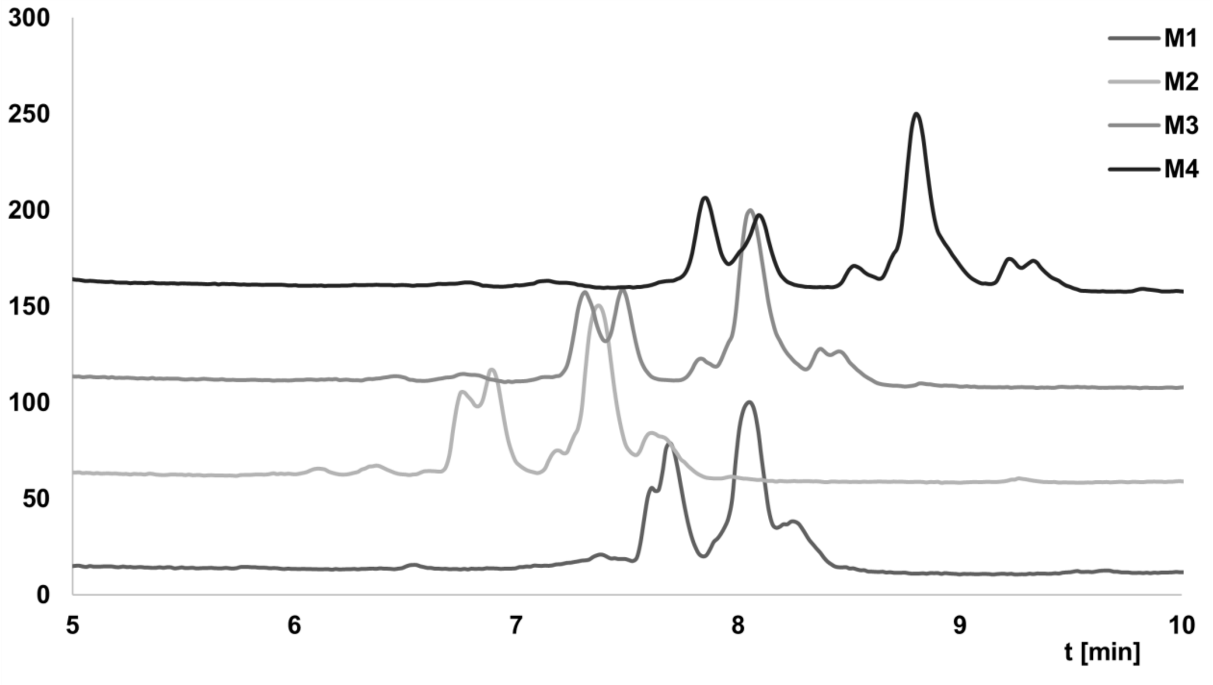

Figure S2. Exemplary TIC separation results of metabolites in the extract obtained from one of the patients after administration of the third nusinersen dose. Notation: M1 – gradient elution: 5-80% MeOH in 15 minutes; M2 – gradient elution: 10-70% MeOH in 15 minutes; M3 – gradient elution: 10-55% MeOH in 15 minutes; M4 – gradient elution: 10-45% MeOH in 15 minutes. Experimental conditions: Kinetex 1.7μm EVO C18 column; mobile phase composition 150mM HFIP/5mM DMBA, MeOH; autosampler temperature 30 °C, column temperature 50 °C; injection volume 2.5 μL; mass spectrometric conditions: nebulizer gas pressure 20 psi, skimmer voltage 60 V, capillary voltage 4000V, fragmentor voltage 250V; drying gas flow 10 l/min; shielding gas flow 10 l/min; octopole voltage 800V; drying gas temperature 350°C; shielding gas temperature 400 °C.

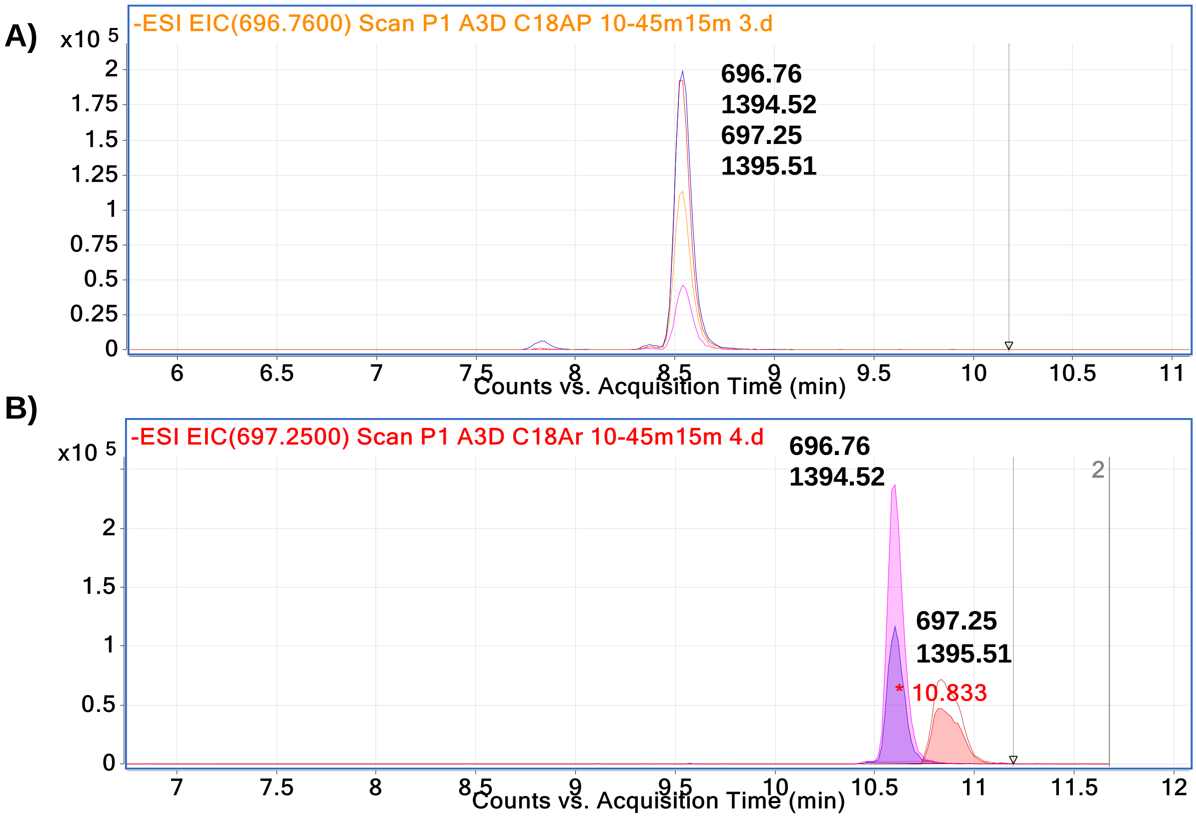

Figure S3. Exemplary EIC chromatograms for two pairs of ions: m/z=696.76 Da, m/z=1394.52 Da and m/z=697.25 Da and m/z=1395.51 Da: A) for C18AP, B) for C18Ar. Experimental conditions: mobile phase composition 150mM HFIP/5mM DMBA, MeOH; gradient elution: 10-45% MeOH in 15 minutes; flow rate 0.3 ml/min; autosampler temperature 30 °C, column temperature 50 °C; injection volume 2.5 µL; mass spectrometric conditions: nebulizer gas pressure 20psi, skimmer voltage 100V, capillary voltage 4000V, fragmentor voltage 250V; drying gas flow 10 l/min; shielding gas flow 10 l/min; octopole voltage 800V; drying gas temperature 350°C; shielding gas temperature 400 °C.

Table S1. Peak areas at extracted ion chromatogram (EIC) obtained for different mass spectrometry parameters and selected ions.

Ion	EIC peak areas												
	Fragmentor voltage [V]				Skimmer voltage [V]			Capillary voltage [V]			Nebulizer gas pressure [psi]		
	100	150	200	250	40	80	100	3000	3500	4000	20	40	60
731.31	76739	157068	515128	553768	594174	774308	788074	527612	608221	772300	782322	701877	670253
1204.55	43474	78252	275568	502615	325322	489927	501585	309899	426569	522874	501093	430053	363381
1307.54	56842	111888	365367	685361	444849	651908	688937	447256	536446	691033	684822	519356	445969
1323.51	23266	59387	159694	305853	199426	254221	308209	129640	238788	310532	319641	252778	206755
1236.48	155347	278248	791835	962254	1039227	1540617	1606259	998477	1401892	1580322	1600315	1302181	972231
1394.55	26839	279890	766193	957919	1015236	1495885	1523436	1069897	1390238	1582009	1618314	1286743	987006
